# The Lixiviation of Metals When Amending Agricultural Soil of the Mediterranean Basin with Biosolids: Trials in Leaching Columns

**DOI:** 10.3390/ijerph192113736

**Published:** 2022-10-22

**Authors:** Manuel M. Jordán, María Belén Almendro-Candel, Ernesto García-Sánchez, José Navarro-Pedreño

**Affiliations:** Department of Agrochemistry and Environment, Miguel Hernández University of Elche, Avd. Universidad s/n, 03202 Elche, Alicante, Spain

**Keywords:** metals, leachate, agricultural soil, biosolids, Mediterranean Basin

## Abstract

An appropriate handling and use of urban and agricultural biosolids on soils are the best means to protect them from erosion, prevent the loss of nutrients due to runoff and washing, and preserve and restore soil productivity. Heavy metal concentrations in biosolids are one of the decisive factors when using this type of waste on soil, due to potentially being harmful to crops and reaching the human food chain. There is a clear need to study the incidence of these metals in agricultural practices in Mediterranean soils. Research for this article was performed as a controlled study using leaching columns. Three treatments were performed by applying different amounts of biosolids (T_50_: 50,000 kg ha^−1^, T_90_: 90,000 kg ha^−1^, T_130_: 130,000 kg ha^−1^), as well as a blank test or control treatment (T0). The presence of macronutrients (K, Na, Ca and Mg), micronutrients (Fe, Cu, Mn and Zn) and three contaminating heavy metals (Cr, Cd and Ni) in lixiviated water was analyzed. Relevant amounts of metals in the wash water were not found. This indicates that, under the watering conditions used, the contaminants and micronutrients analyzed are not a relevant source of water contamination on a common calcareous soil of the Mediterranean Basin.

## 1. Introduction

Adding biosolids on or under the soil’s surface is probably the most common way of applying them. Biosolids are one of the most common materials used to organically amend soils [1]. In some states of the USA, such as Colorado, Florida, Oregon and Washington, 76% of biosolids generated are used in agriculture [1]. Sunlight, soil microorganisms and drying help destroy pathogens and numerous toxic organic substances in sewage sludge. The elements that make up the soil react with heavy metals in biosolids and nutrients. These reactions help immobilize the metals, thus decreasing their contaminating potential [2]. However, a program to apply biosolids to agricultural soils must consider several factors. These include: (i) the characteristics of the soil, such as the water table, slope, soil permeability, mineralogy, pH and public access, and (ii) the doses of biosolid applied, which are mainly determined by the concentration of nutrients, heavy metals or toxic organic substances in the biosolid [3].

Dehydrated biosolids are often applied to the surface and can be mixed with the surface soil horizon by tilling. The techniques used to apply dehydrated biosolids are basically the same as when manuring, with machinery that spreads it over the surface. The need for tilling to ensure a thorough mix with the soil limits its use in forest soils or on tricky terrain. Liquid biosolids can be injected in the soil or sprayed or spread over the surface. Injecting liquid sewage sludge in the subsurface is a relatively new technique. It is gaining traction because the biosolid is covered by the soil, which improves its appearance and helps hide the smell. The injection technique is also safe for applying suspensions of waste from farms and livestock farms, as the physical characteristics of the biosolid and these suspended substances are similar. The potential benefit of injection over surface application methods is that the liquid is buried under the surface. Even though this requires more equipment and energy, it decreases the smells, pathogens [4], insect plagues and losses due to runoff [5], along with the possibility of direct contact between the biosolid and plants, animals or humans. Soil injection also removes the surface runoff in soils with a steep slope. Soils that cannot receive surface sludge due to their proximity to human communities are also prime candidates for the injection process.

An appropriate handling and use of urban and agricultural organic waste on soils are the best means to protect them from the erosion caused by wind and water, prevent the loss of nutrients due to runoff and washing, and preserve and restore soil productivity [6]. Organic materials are not only used as sources of nutrients, but also as a soil conditioner, as they improve the physical properties of the soil, increase water infiltration, water-retaining capabilities, aeration, permeability, soil aggregation and root depth, while decreasing runoff and erosion [7].

When the concentrations of heavy metals surpass the recommended levels in certain biosolids, it is generally due to the way in which the metals are being dumped in the sewage systems by industries. Therefore, we must be more careful when using this waste in agricultural soil. When treating wastewater, 70–90% of the metals are transferred to the biosolid by adsorption and precipitation [4]. Furthermore, when applied to soils, the bioavailability of metals attached to the biosolid is affected by the properties of the soil (e.g., pH, clay and Fe and Al hydroxide content, organic matter) and by the number of metals added [8]. In general, the total amount of heavy metals is higher in clay soils, but their availability for plants can be low in these soils due to the resistance that keeps these metals in clay surfaces [9].

From the viewpoint of plant nutrition, heavy metals fall into two categories:

- Essential elements: Cu, Fe, Mn, Mo and Zn. Plants need small amounts, but higher or lower concentrations lead to toxicity or deficits that prevent their normal development.
- Non-essential elements: Cd, Hg, Pb, Ni and Cr. Plants tolerate low concentrations well and do not show symptoms of toxicity until certain concentration limits are surpassed [10].

Heavy metal concentrations in the biosolid are one of the decisive factors when using this type of waste on soil, due to potentially being harmful to crops and reaching the human food chain [11].

The close connection between agriculture and the environment often makes it hard to differentiate between effects caused by human activities and the area’s natural evolution. There is a clear need to study the impact of agricultural practices. This is the purpose of this study, which aims to produce experimental knowledge on a specific situation that is common on agricultural soils of the Mediterranean Basin and is present in our surroundings. The use of waste in agriculture is leading to the addition of agents to the environment that could induce improvements and increase productivity—as well as contamination issues [1,11,12].

Soil is located at the point where the atmosphere and hydrosphere come into contact. This area is a key piece of the development of the biosphere and a crucial part of the water cycle. Therefore, dumping waste in the environment can cause issues connected to the quality of soils and water [13]. The use of biosolids in agriculture can be a guarantee of success in soil productivity, but it is important to preserve the conservation of the environment with less risk of contamination of surface and groundwater. Many physical and chemical properties in soils amended with biosolid, such as water retention capacity, aggregate stability, contribution of N, P and other nutrients to crop growth, depend, to some extent, upon the quantity of organic matter in the biosolid that is added.

These premises and the condition of the soil of the Mediterranean Basin (calcareous and with limited organic matter) lead to the novelty and the objectives of this study being: (i) to define the lixiviation behavior of biosolids from wastewater treatment that are used to amend calcareous soil, and (ii) to determine the mobility of essential and heavy metals present in the soil through the profile.

## 2. Materials and Methods

### 2.1. Soil Characterisation

**Figure 1:**
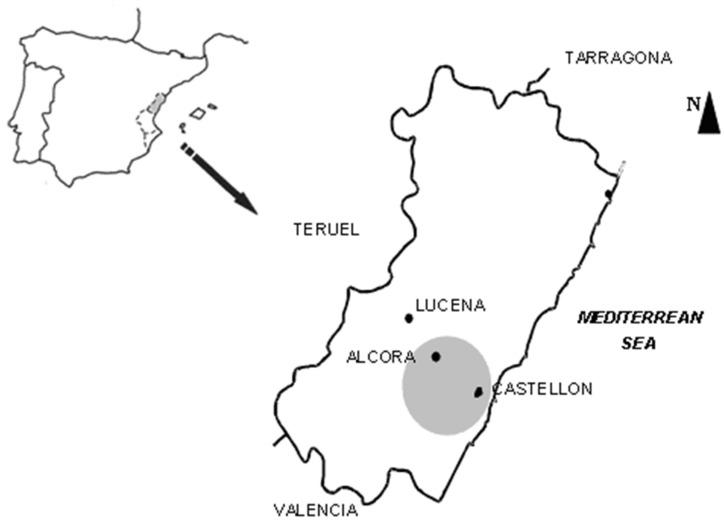
Location of the area studied (Scale 1:2,000,000).

The soil used to carry out this experience was an agricultural soil from an area located just north of the town of Castellón, NE Spain (Figure 1).

The main properties of the soil chosen for this study are shown on Table 1. The soil had a basic pH and would therefore have an alkaline reaction. This entails that there could be issues with the availability of most nutrients, and that we could have to make acidic amendments using biosolids to decrease the pH, facilitate the mobility of the elements and improve the soil’s structure.

The amount of equivalent calcium carbonate was high, as traditionally occurs with soils of the Spanish Mediterranean coast. The amount of organic matter was very low compared to the normal amount expected for farmland soil, which should be between 30 and 50 g/kg. Regarding the amount of assimilable nutrients (extracted with ammonium acetate and DTPA), all elements were present in low or very low levels, except for Ca.

### 2.2. Biosolid Characterisation

Regarding the biosolid applied in this study, we used sewage sludge from a wastewater treatment plant located in the l’Alcalatén district (Alcora, Castellón, Spain), at the heart of a ceramic cluster. The composition of this biosolid is shown in Table 2.

### 2.3. Teatments and Irrigation: Trials in Columns

To conduct the research, we performed a controlled study using leaching columns that met OECD (2004) guidelines [14]. To do so, we cut a PVC pipe with 10.5 cm of inner diameter into 20-centimetre-long pieces and created 80-centimetre-high columns with them. Several authors [11,15,16,17] have conducted and validated similar trials. For each treatment, three replicates were done. Three treatments were performed by applying different amounts of biosolids (T_50_: 50,000 kg ha^−1^, T_90_: 90,000 kg ha^−1^, T_130_: 130,000 kg ha^−1^), as well as a blank test or control treatment (T_0_). The biosolid was applied to the surface and mixed with the soil to simulate tilling, producing a homogenous mixture of biosolid with the first 20 cm of soil. In order to establish the closest parallels between real conditions and those of the experiment, the soil contained in the columns was irrigated using tap water. The irrigation applications lasted eight months. Collection of the leached water was carried out 24 h after the last application. This irrigation is equivalent to a weekly rainfall of 100 mm. The contribution of water was provided by a device that simulated short rainfall or a flood irrigation system that covered the surface and then percolated into the soil. It consists of a plastic recipient with holes punched in the bottom.

To establish the highest possible level of similarity between real conditions and the experiment, every seven days we supplied the soil in the columns an amount of water that equated to 75 mm of rain a week. This is similar to the total amount of irrigation water provided when growing vegetables. The water was supplied through a device that simulated short bouts of rain or a flood irrigation system whereby the water covered the surface and then seeped into the soil. Sampling was carried out four times during the study, leaving two months between each one. Leached water was collected during each round of sampling in addition to the soil from the columns.

### 2.4. Leachates: Chemical and Statistical Analysis

Ions Na^+^, K^+^, Mg^2+^ and Ca^2+^ were measured directly in the sample or in suitable dilutions. The first two using atomic emission spectrophotometry, and the last two through atomic absorption spectrophotometry. Mn, Fe, Cu, Zn, Ni, Cd and Cr were measured using an atomic absorption technique with a graphite chamber (Agilent PSD 100/120 GFAAS, Santa Clara, CA, USA).

Knowing whether the treatments caused significant differences in the parameters analysed requires a statistical assessment. In this sense, a statistical analysis can be used to determine the level of confidence of the results obtained. The statistical treatment applied to this study’s data is based on an analysis of variance (ANOVA) with a single classification criterion to verify the hypothesis of equality between population means [18]. Symbols *, ** and *** indicate significance at levels of *p* = 0.05, 0.01 and 0.001 respectively, while ‘ns’ stands for ‘not significant’ in tables [19].

## 3. Results and Discussion

### 3.1. Potassium, Sodium, Calcium and Magnesium

The concentrations of these elements in leachates do not pose a risk from the viewpoint of water for human consumption. Regarding potassium, only the second sampling showed a significant increase with treatment. However, the last sampling shows the opposite tendency (Table 3). The soluble K added with the sludge does not seem to increase this element in leachates. The clayey nature of this soil (39%) may limit the movement and loss of this nutrient, which binds relatively easily to clays. The biosolid provided NH^4+^, which could compete with K^+^ for binding sites. There seem to be enough exchange sites in the soil colloids to prevent significant losses due to washing.

The first sampling shows a clear increase in sodium as a result of applying increasing amounts of biosolids. Increases are not significant in other samplings (Table 4). It tends to lixiviate more over time.

This cation does not bind as easily as K^+^ and is thus more easily washed out. Neither K nor Na seems to depend on the mineralization processes of the biosolid’s organic fraction. They must be linked to the inorganic fraction (precipitating as salts, mainly sulphates and carbonates).

Calcium seems to increase with the treatment (Table 5). Over time, it tends to decrease, as the last sampling shows no difference between the control group and the lowest level of treatment (T50).

The soil’s reaction with the biosolid, the possible acidic attack on the carbonates, seems to have partly increased the amount of soluble Ca transported by the wash water, as it appears in significant amounts in the leachates. In the soil’s aqueous extract, the most noticeable differences were found in the surface horizon.

Magnesium increases significantly with the treatment and decreases over time (Table 6). It behaves similarly to calcium, and, in this soil, it is chemically characterized by having carbonates.

### 3.2. Iron, Manganese, Copper and Zinc

The concentrations of metals like iron, manganese, copper and zinc in water are usually very low, except in certain cases where the water’s origin entails higher metal concentrations. None of these elements were found in the leachates in concentrations higher than those established for local drinking water.

Iron increases significantly with treatment (Table 7). It is worth noting that the biosolid’s heightened amount of Fe caused a displacement of the metal to the wash water.

Manganese in the leachate tends to increase with increasing amounts of supplied biosolids and decrease over time, as there was the same concentration for all treatments in the last sampling (Table 8). The sludge does not have such a big impact in this case as with the iron.

Copper seems to increase with the treatment and over time (Table 9). This element is connected to the organic fraction. The mineralization and decrease in the latter could explain the micronutrient’s increase in mobility and loss by lixiviation.

Zinc behaves like iron, increasing with the treatment and over time (Table 10). In this case, the biosolid has as much of an impact as with the iron.

### 3.3. Cadmium, Chromium and Nickel

The treatment only altered the cadmium in the third sampling, where it increased when applying the biosolid (Table 11). In the other samplings, there are no significant differences between the control and the treatment groups. The presence of compounds with low molecular weight—from the breakdown of organic matter in all treatment groups—can favor the displacement of Cd to the wash water. In addition, some authors have found that the presence of Zn favors the lixiviation of Cd, creating compounds with ligands such a Cl^−^, as both are more mobile throughout the soil’s profile [20].

The concentration of chromium increases in leachates when applying sludge (Table 12). A higher presence of Cr in water coincides with increased Cd. It seems like the breakdown of the soil’s organic matter had an impact in both cases. One concern is the lack of analysis of metal speciation, especially the chromium speciation analysis. Trivalent chromium is non-toxic and is under dispute regarding its essentiality for humans. Hexavalent chromium is highly toxic for plants and animals [21,22,23,24]. The limitation of this study in terms of metal speciation is evident.

Nickel increases when applying biosolid and decreases over time. There are no differences between the control and treatment groups in the last sampling (Table 13). In this case, the metal initially has greater mobility than in the two previous samplings.

An exhaustive analysis of the literature, it can be concluded that there are few recent studies that address this environmental problem and that the results obtained are very different. Researchers [25] found that Ni and Cd tend to remain in the biosolid incorporation zone for a period of nine years. After applying biosolids to the grassland surface, other researchers [26] discovered that Cr, Ni and Cd were moving towards the top 10 cm of the profile. Some authors [27] found that most of the heavy metals were limited to the first 7 cm of the surface in forest soils. However, there are other authors [28] who found a higher movement of heavy metals. In these studies, on soils that had been repeatedly amended with biosolids, Ni moved from the topsoil to depths of 40–60 cm, Pb to depths of 20–40 cm and Cd to depths of 60–80 cm. However, the Cr remained on the surface. In a recent study in 2019, a similar behavior for Ni and Cr was reported [29]. In our study, there are extremely low concentrations of these three contaminating metals in the leachates, all remaining below the maximum levels allowed for drinking water. Therefore, their mobility is not important in this Mediterranean calcareous soil. This was also suggested by the low extractability of these metals in water in the soil analyzed.

## 4. Conclusions

Under the watering conditions used, high amounts of metals did not appear in wash water related to the low mobility of toxic ions in a peculiar studied calcareous Mediterranean soil (pH > 8) with limited organic matter. This indicates that, under the watering conditions used, the heavy metals and micronutrients studied are not a source of contamination of the relevant surface and underground waters. This initial interpretation of the results helps obtain an overview of what happens in this calcareous soil when adding wastewater biosolid and discovers the issues that said fertilization can entail. Subsequent, more detailed studies using the values obtained herein may allow us to gain a deeper understanding of the connections between the properties of the soil, the characteristics of the waste and the behavior of the various fractions of nutrients and contaminants analyzed in the soil and leachates. This is undoubtedly a starting point to conduct a mathematical study that makes it possible to model and assess the evolution and mobility of these elements in the soil. This work and similar experiments with other types of soils could help obtain more general conclusions and correlations between different variables that optimize the decision-making of soil managers. This paper is a small contribution to the study of the mobility of metals in agricultural soils of the Mediterranean Basin. There is certainly a long way to go in this subject in the EU. Some authors in 2016 [30] carried out a detailed analysis of the heavy content in agricultural topsoils of the European Union (EU). These authors estimated that more than 6% or 137,000 km^2^ of agricultural soils in the EU need local assessment and eventual remediation action and suggested the establishment of harmonized screening values for soil contamination in the EU [31].

## Figures and Tables

**Table 1:** Characteristics of the soil used in the study.

Parameter		Value	Parameter		Value
Texture				Mineralogy	
Sand 20 < ∅ < 2000 μm	%	26	Soil	Quartz	Calcite
Silt 2 < ∅ < 20 μm	%	35	Clay Fraction	Quartz	Illite
Clay < ∅ < 2 μm	%	39	Total elements		
pH		8.02	Al	g/kg	20.1
EC	μS/cm	77.7	B	mg/kg	39.1
Oxidisable C	g/kg	1.8	Ca	g/kg	226.2
Org. M. Oxid.	g/kg	2.9	Cd	μg/kg	228
N Kjeldhal	g/kg	0.6	Cr	mg/kg	20.9
P	mg/kg	16.70	Fe	g/kg	12.8
Extracted ammonium acetate			K	g/kg	4.2
Ca	g/kg	5.342	Li	mg/kg	9.1
K	g/kg	0.149	Mg	g/kg	4.9
Mg	g/kg	0.256	Mn	mg/kg	170.2
Na	g/kg	0.069	Mo	mg/kg	1.9
Extracted DTPA (diethylenetriaminepentaacetic acid)			Na	g/kg	0.490
Cu	mg/kg	0.33	Ni	mg/kg	19.2
Mn	mg/kg	0.98	Sr	mg/kg	39.83
Zn	mg/kg	0.22	Zn	mg/kg	33.5

**Table 2:** Biosolid composition (dry matter).

Parameter	Unit	Value	Parameter	Unit	Value
Humidity	%	83	Oxidisable C	%	20.2
Org M. 500 °C	%	61.2	Org. Oxid. M.	%	34.1
Al	g/kg	12.23	Mg	g/kg	5.69
As	mg/kg	1.0	Mn	mg/kg	153
Ba	mg/kg	539	N	g/kg	42.15
Ca	g/kg	58.02	Na	g/kg	9.75
Cd	mg/kg	39.1	Ni	mg/kg	292
Cr	mg/kg	29	P	mg/kg	2375
Cu	mg/kg	415	Pb	mg/kg	89
Fe	g/kg	42.35	B	mg/kg	4
Hg	μg/kg	1.6	Ti	mg/kg	20
K	g/kg	1.47	Zn	mg/kg	2163

**Table 3:** Potassium content (mg/L) in leachates. Entries represent means ± standard errors from *n* = 3 replicates.

Treatment	Sampling			
	60 Days	80 Days	100 Days	120 Days
T_0_	1.6 ± 0.1	1.5 ± 0.3	1.0 ± 0.55	1.2 ± 1.05
T_50_	1.3 ± 0.2	1.8 ± 0.25	1.1 ± 0.75	1.0 ± 0.4
T_90_	1.4 ± 0.25	2.3 ± 0.65	1.1 ± 0.45	0.9 ± 0.5
T_130_	1.5 ± 0.3	2.3 ± 0.4	1.2 ± 0.5	0.4 ± 0.45
F-ANOVA	2.05 ^ns^	7.29 *	0.20 ^ns^	0.35 ^ns^

**Table 4:** Sodium content (mg/L) in leachates. Entries represent means ± standard errors from *n* = 3 replicates.

Treatment	Sampling			
	60 Days	80 Days	100 Days	120 Days
T_0_	19 ± 3	20 ± 2	132 ± 12	24 ± 3
T_50_	21 ± 2	22 ± 4	28 ± 14	32 ± 15
T_90_	24 ± 5	25 ± 7	32 ± 23	33 ± 11
T_130_	28 ± 6	26 ± 9	29 ± 5	30 ± 14
F-ANOVA	18.15 ***	4.06 ^ns^	0.07 ^ns^	2.21 ^ns^

**Table 5:** Calcium content (mg/L) in leachates. Entries represent means ± standard errors from *n* = 3 replicates.

Treatment	Sampling			
	60 days	80 days	100 days	120 days
T_0_	122 ± 19	121 ± 69	96 ± 20	99 ± 25
T_50_	139 ± 65	142 ± 28	110 ± 41	98 ± 17
T_90_	181 ± 15	119 ± 22	139 ± 58	139 ± 39
T_130_	152 ± 59	135 ± 31	160 ± 11	121 ± 23
F-ANOVA	7.18 *	1.17 ^ns^	12.07 **	6.58 *

**Table 6:** Magnesium content (mg/L) in leachates. Entries represent means ± standard errors from *n* = 3 replicates.

Treatment	Sampling			
	60 Days	80 Days	100 Days	120 Days
T_0_	10.5 ± 2.6	9.7 ± 1.8	6.9 ± 1.8	8.5 ± 5.0
T_50_	12.5 ± 4.2	12.3 ± 2.0	7.8 ± 1.7	7.9 ± 3.2
T_90_	13.5 ± 2.4	11.9 ± 1.9	10.7 ± 0.6	11.0 ± 3.4
T_130_	14.7 ± 1.8	13.0 ± 4.1	12.9 ± 1.2	9.9 ± 1.8
F-ANOVA	6.53 *	4.05 ^ns^	69.03 ***	3.77 *

**Table 7:** Iron content (μg/L) in leachates. Entries represent means ± standard errors from *n* = 3 replicates.

Treatment	Sampling			
	60 Days	80 Days	100 Days	120 Days
T_0_	2.5 ± 0.2	2.8 ± 1.0	1.3 ± 1.6	1.1 ± 0.4
T_50_	3.9 ± 1.8	3.4 ± 1.2	1.8 ± 1.4	2.7 ± 0.8
T_90_	4.9 ± 2.4	7.1 ± 5.1	3.7 ± 2.9	4.6 ± 2.9
T_130_	3.4 ± 1.2	6.2 ± 1.3	4.4 ± 2.0	7.9 ± 1.7
F-ANOVA	4.01 ^ns^	12.04 **	11.36 **	54.49 ***

**Table 8:** Manganese content (μg/L) in leachates. Entries represent means ± standard errors from *n* = 3 replicates.

Treatment	Sampling			
	60 Days	80 Days	100 Days	120 Days
T_0_	0.2 ± 0.1	0.2 ± 0.1	0.2 ± 0.1	0.1 ± 0.0
T_50_	0.3 ± 0.1	0.3 ± 0.1	0.2 ± 0.0	0.1 ± 0.0
T_90_	0.2 ± 0.3	0.4 ± 0.1	0.2 ± 0.0	0.1 ± 0.1
T_130_	0.6 ± 0.1	0.4 ± 0.2	0.3 ± 0.2	0.2 ± 0.1
F-ANOVA	5.06 *	7.61 **	6.72 *	1.20 ^ns^

**Table 9:** Copper content (μg/L) in leachates. Entries represent means ± standard errors from *n* = 3 replicates.

Treatment	Sampling			
	60 Days	80 Days	100 Days	120 Days
T_0_	0.8 ± 0.7	0.7 ± 0.5	1.5 ± 1.0	1.8 ± 0.8
T_50_	1.3 ± 0.7	1.2 ± 0.8	3.2 ± 0.7	1.6 ± 0.6
T_90_	1.5 ± 0.6	1.1 ± 0.5	2.9 ± 2.0	2.6 ± 0.9
T_130_	1.1 ± 0.3	1.3 ± 0.3	3.0 ± 1.9	3.8 ± 1.8
F-ANOVA	1.08 ^ns^	8.61 **	6.02 *	16.10 **

**Table 10:** Zinc content (μg/L) in leachates. Entries represent means ± standard errors from *n* = 3 replicates.

Treatment	Sampling			
	60 Days	80 Days	100 Days	120 Days
T_0_	0.1 ± 0.0	0.3 ± 0.2	1.6 ± 0.2	1.0 ± 1.0
T_50_	0.6 ± 1.5	1.9 ± 1.3	3.6 ± 1.6	2.7 ± 0.9
T_90_	1.1 ± 0.6	3.1 ± 1.4	5.8 ± 2.9	5.9 ± 1.2
T_130_	3.2 ± 2.8	6.2 ± 1.3	10.3 ± 0.4	12.2 ± 7.8
F-ANOVA	7.60 *	85.20 ***	112.01 ***	27.21 ***

**Table 11:** Cadmium content (μg/L) in leachates. Entries represent means ± standard errors from *n* = 3 replicates.

Treatment	Sampling			
	60 Days	80 Days	100 Days	120 Days
T_0_	0.2 ± 0.1	0.2 ± 0.0	1.3 ± 0.3	0.9 ± 0.1
T_50_	0.2 ± 0.2	0.1 ± 0.0	1.2 ± 0.2	0.8 ± 0.2
T_90_	0.2 ± 0.1	0.1 ± 0.1	1.7 ± 0.3	0.8 ± 0.2
T_130_	0.2 ± 0.2	0.2 ± 0.2	4.7 ± 0.9	0.9 ± 0.3
F-ANOVA	0.59 ^ns^	1.76 ^ns^	78.16 ***	0.12 ^ns^

**Table 12:** Chromium content (μg/L) in leachates. Entries represent means ± standard errors from *n* = 3 replicates.

Treatment	Sampling			
	60 Days	80 Days	100 Days	120 Days
T_0_	0.6 ± 0.2	0.2 ± 0.5	0.6 ± 0.7	0.2 ± 0.2
T_50_	0.8 ± 0.2	0.6 ± 0.1	0.8 ± 0.3	1.0 ± 0.1
T_90_	0.9 ± 0.4	0.3 ± 0.1	0.9 ± 0.4	1.1 ± 0.4
T_130_	1.0 ± 0.9	0.4 ± 0.3	1.6 ± 0.6	1.2 ± 0.6
F-ANOVA	2.02 ^ns^	1.81 ^ns^	11.95 **	11.98 **

**Table 13:** Nickel content (μg/L) in leachates. Entries represent means ± standard errors from *n* = 3 replicates.

Treatment	Sampling			
	60 Days	80 Days	100 Days	120 Days
T_0_	0.7 ± 0.5	0.4 ± 0.1	0.2 ± 0.3	0.4 ± 0.2
T_50_	1.2 ± 0.1	1.1 ± 0.4	0.3 ± 0.4	0.2 ± 0.3
T_90_	1.4 ± 0.6	0.7 ± 0.6	0.8 ± 0.1	0.3 ± 0.2
T_130_	1.9 ± 0.4	1.6 ± 1.0	1.1 ± 0.5	0.5 ± 0.1
F-ANOVA	22.99 ***	5.41 *	26.08 ***	1.26 ^ns^

## Data Availability

Data available on request from the authors.
